# Downregulation of the Canonical WNT Signaling Pathway by TGFβ1 Inhibits Photoreceptor Differentiation of Adult Human Müller Glia with Stem Cell Characteristics

**DOI:** 10.1089/scd.2015.0262

**Published:** 2015-10-11

**Authors:** Angshumonik Angbohang, Na Wu, Thalis Charalambous, Karen Eastlake, Yuan Lei, Yung Su Kim, Xinghuai H. Sun, G. Astrid Limb

**Affiliations:** ^1^ Division of Ocular Biology and Therapeutics, UCL Institute of Ophthalmology, London, United Kingdom.; ^2^ Department of Ophthalmology and Visual Science, Eye & ENT Hospital, Shanghai Medical College, Fudan University, Shanghai, China.; ^3^ NIHR Biomedical Research Centre for Ophthalmology at Moorfields Eye Hospital and UCL Institute of Ophthalmology, London, United Kingdom.

## Abstract

Müller glia are responsible for the retina regeneration observed in zebrafish. Although the human retina harbors Müller glia with stem cell characteristics, there is no evidence that they regenerate the retina after disease or injury. Transforming growth factor-β (TGFβ) and Wnt signaling regulate retinal neurogenesis and inflammation, but their roles in the neural differentiation of human Müller stem cells (hMSC) are not known. We examined hMSC lines in vitro for the expression of various Wnt signaling components and for their modulation by TGFβ1, as well as the effect of this cytokine on the photoreceptor differentiation of these cells. Culture of hMSC with a combination of factors that induce photoreceptor differentiation of hMSC (FGF2, taurine, retinoic acid, and insulin-like growth factor type1; FTRI), markedly upregulated the expression of components of the canonical Wnt signaling pathway, including WNT2B, DKK1, and active β-CATENIN. Although FTRI did not modify mRNA expression of *WNT5B*, a component of the noncanonical/planar cell polarity Wnt pathway, it upregulated its secretion. Furthermore, TGFβ1 not only decreased *WNT2B* expression, but also inhibited FTRI-induced photoreceptor differentiation of hMSC, as determined by expression of the photoreceptor markers NR2E3, RHODOPSIN, and RECOVERIN. Inhibition of TGFβ1 signaling by an ALK5 inhibitor prevented TGFβ1-induced changes in the expression of the two Wnt ligands examined. More importantly, inhibition of the canonical WNT signaling by XAV-939 prevented FTRI-induced photoreceptor differentiation. These observations suggest that TGFβ may play a key role in preventing neural differentiation of hMSC and may constitute a potential target for induction of endogenous regeneration of the human retina.

## Introduction

The spontaneous retinal regeneration observed in zebrafish has been ascribed to the ability of a population of Müller glia to dedifferentiate and become progenitors that give rise to retinal neurons [1]. Although Müller glia dedifferentiation into retinal progenitors has not been demonstrated in vivo in the human eye, a population of Müller glia able to proliferate indefinitely in vitro has been identified [2]. Unlike their inability to regenerate the human retina, when cultured with selective growth and differentiation factors these cells can be induced to acquire characteristics of retinal neurons, for which they have been termed human Müller stem cells (hMSC) [2–5]. The reasons why Müller glia do not regenerate the adult human retina are not known, but it is possible that factors produced in adult life or during degenerative diseases may prevent these cells from exerting these functions in vivo. Most retinal degenerative conditions that lead to blindness, including inflammatory, proangiogenic, and dystrophic retinal diseases, have been associated with abnormal proliferation of Müller glia that does not lead to repair, but to the formation of glial scarring [6]. Many of these conditions are also accompanied by local increased production of proinflammatory cytokines, such as transforming growth factor-β (TGFβ) [7–9], which may potentially modify the neural progenicity of hMSC.

TGFβ signaling mediated through Smad 2/3, which is controlled by transcriptional corepressors such as Tgif1 and Six3b, has been demonstrated to regulate Müller glia-derived photoreceptor regeneration in the adult zebrafish [10]. In addition, signaling by the TGFβ superfamily (including TGFβ1, TGFβ2, TGFβ3, and bone morphogenic proteins) regulates mammalian cell proliferation, differentiation, migration, and apoptosis during embryogenesis [11]. During early development, TGFβ has been shown to synergize or antagonize with Wnt proteins, a family of highly conserved secreted signaling molecules that regulate cell-to-cell interactions [12–14]. Wnt signaling is a major regulator of neurogenesis in the adult hippocampus [15], and it has been suggested that the pathways initiated by various Wnt ligands may depend on the receptors expressed by a given target cell [16]. Activation of the canonical Wnt signaling by TGFβ has been shown to mediate fibrosis [17], and cooperation between TGFβ and Wnt signaling pathways are known to play a role in controlling developmental events such as the regulation of osteoblast differentiation of human mesenchymal stem cells [18]. In addition, it has been shown that Wnt signaling is activated during retina degeneration and that Wnt activation protects retinal cells from oxidative stress. From these observations it is suggested that Wnt activation by growth factors may increase the threshold for apoptosis and prevent further photoreceptor degeneration [19].

Although interaction of these signaling pathways in fish and amphibians as well as small mammals during development and adult regeneration are documented [20], there is no knowledge of the role of these factors in the regulation of neural differentiation of hMSC in the adult human eye. On this basis, we investigated the role of TGFβ1 on the regulation of the WNT signaling pathway in hMSC by examining the effect of this cytokine on the expression of the DKK1 and WNT2B and WNT5B ligands, previously shown to be expressed by mammalian Müller glial cells [19], as well as on the phosphorylation of β-catenin in cells undergoing photoreceptor differentiation. In this study, we demonstrate that TGFβ1 downregulates the expression of the canonical Wnt signaling ligand WNT2B and increases the phosphorylation of β-catenin, while upregulating the expression of the noncanonical Wnt ligand WNT5B in hMSC. Interestingly, TGFβ1 inhibits photoreceptor differentiation of hMSC in vitro, an event shown to be mediated by activation of the canonical Wnt signaling pathway.

## Materials and Methods

### Human Müller stem cell culture

Human Müller stem cell lines (hMSC) developed in our laboratory were cultured as previously described [2]. Briefly, cells were grown in Dulbecco's modified Eagle's medium (DMEM) (Gibco-BRL) containing 10% fetal calf serum (FCS) and 1% Penicillin/Streptomycin mix. Three different hMSC, named MIO-M1, MIO-M7, and MIO-M8, from passages 7 to 33 were used for the experiments. To assess the effect of TGFβ1, cells were cultured for 7 days in the presence or absence of human recombinant TGFβ1 (PeproTech) at log_10_ concentrations ranging from 0.1 to 100 ng/mL. To investigate the effect of TGFβ1 on the neural differentiation of hMSC, cells were cultured for 7 days in flasks coated with basement membrane protein (bMP) (Sigma-Aldrich) in the presence or absence of TGFβ1 (50 ng/mL) and factors known to induce the expression of photoreceptor cell markers as previously published by our group [3]. Briefly, to induce photoreceptor differentiation, cells were cultured for 5–6 days in bMP-coated flasks in the presence of 20 ng/mL FGF2, 20 μM taurine (Sigma Aldrich), 5 μM retinoic acid (Sigma Aldrich), and 100 ng/mL IGF-1 (PeproTech) (FTRI) [3]. In selected experiments, hMSC were stimulated with recombinant human TGFβ1 (PeproTech) alone, or in combination with inhibitors of the TGFβ pathway or β-catenin at the following concentrations of 10 μM TGFβ type I (ALK5) receptor inhibitor SB431542 (Selleckchem), 20 μM JNK inhibitor SP600125 (Sigma-Aldrich), and 10 nM β-CATENIN inhibitor XAV-939 (Selleckchem). Furthermore, to investigate the effect of WNT2B and WNT5B on the expression of DKK1, hMSC were cultured with these recombinant proteins for 7 days at concentrations of 100 ng/mL (WNT2B; Abnova) and 500 ng/mL (WNT5B; R&D Systems).

### RNA isolation and reverse transcription–polymerase chain reaction

Total RNA was isolated using the RNeasy Mini Plus Kit (Qiagen) according to the manufacturer's instructions. Quantification of total RNA was performed using a NanoDrop spectrophotometer (Thermo Scientific). Reverse transcription (RT) was performed using 1 μg RNA as per the manufacturer's instructions (Life technologies). Polymerase chain reaction (PCR) was then performed using the primer sequences shown in Table 1. Amplification was performed in a 20 μL volume by mixing 10 μL of 2× Green GoTaq Mix (Promega), 1 μL of 10 μM primers, and 0.5–1 μL cDNA. Final volume was adjusted with RNAse-free water (Promega). The instrumental settings were as follows: initial denaturation step of 2 min at 95°C, followed by 26–34 cycles as follows: denaturation 94°C for 30 s, annealing temperature for 30 s and extension 72°C for 30 s, and one cycle of 72°C for 5 min. PCR products were then analyzed by agarose gel electrophoresis (2%) containing 10,000 × GelRed Nucleic Acid Stain (Biotium).

**Table 1.** Primer Sequences Used for RT-PCR to Assess Gene Expression of Components of the Wnt Signaling Pathway and Photoreceptor Differentiation Markers in hMSC

*Gene*	*Accession No.*	*Sequence*	*Tm(*°*C)*	*Product size(bp)*
*β-ACTIN*	NM_001101	(F) CATGTACGTTGCTATCCAGGC	60	250
		(R) CTCCTTAATGTCACGCACGAT		
*WNT2B*	NM_004185.3	(F) GACGGCAGTACCTGGCATAC	58	188
		(R) CTCCTTAATGTCACGCACGAT		
*WNT3A*	NM_033131.3	(F) AGATGGTGGTGGAGAAGCAC	58	290
		(R) GTAGCAGCACCAGTGGAACA		
*WNT5B*	NM_032642.2	(F) TTCTGACAGACGCCAACTC	58	264
		(R) TGACTCTCCCAAAGACAGATG		
*WNT8B*	NM_003393.3	(F) CCATGAACCTGCACAACAA	58	174
		(R) TGAGTGCTGCGTGGTACTTC		
*WNT11*	NM_004626.2	(F) TGACCTCAAGACCCGATACC	58	214
		(R) GCTTCCGTTGGATGTCTTGT		
*FZD1*	NM_003505.1	(F) AGACCGAGTGGTGTGTAATGA	60	253
		(R) ATACTGTGAGTTGGCTTCGAT		
*FZD4*	NM_012193	(F) AACTTTCACACCGCTCATC	55	391
		(R) CAGCATCATAGCCACACTTG		
*FZD5*	NM_003468.3	(F) TTCTGGATAGGCCTGTGGTC	60	214
		(R) CGTAGTGGATGTGGTTGTGC		
*FZD7*	NM_003507	(F) GCTCTTTACCGTTCTCACCTA	55	388
		(R) CAGGATAGTGATGGTCTTGAC		
*β-CATENIN*	NM_001904.3	(F) TACCTCCCAAGTCCTGTATGAG	60	180
		(R) TGAGCAGCATCAAACTGTGTAG		
*DKK1*	NM_012242.2	(F) CCTTGAACTCGGTTCTCAATTCC	60	138
		(R) CAATGGTCTGGTACTTATTCCCG		
*RHODOPSIN*	NM_000539	(F) GCTTCCCCATCAACTTCCTCA	60	156
		(R) AGTATCCATGCAGAGAGGTGTAG		
*NR2E3*	NM_014249.3	(F) GGCGTGGAGTGAACTCTTTC	58	230
		(R) CTGGCTTGAAGAGGACCAAG		
*RECOVERIN*	NM_002903	(F) AGCTCCTTCCAGACGATGAA	60	150
		(R) CAAACTGGATCAGTCGCAGA		

hMSC, human Müller stem cells; RT-PCR, reverse transcription-polymerase chain reaction.

### Protein analysis

#### Western blotting

Cell lysates were prepared using RIPA buffer (Thermo Scientific) containing protease inhibitor cocktail (Sigma). Gels and buffer systems (NuPAGE; Invitrogen) were used for western blot analysis, as previously described [4,21] Briefly, 24 μL of loading sample were prepared with 10 μg protein, 2.4 μL reducing agent (10×), 6 μL loading buffer (LDS 4×), and water. Proteins were denatured at 80°C for 10 min before loading onto 4%–12% Bis-Tris gels. Gels were run using the MOPS buffer containing antioxidant at 180 V for 60 min, after which they were semi-dry transferred onto polyvinylidene difluoride membranes (Millipore) at 10 V for 46 min. Membranes were blocked in TBS +0.1% Tween-20 containing 5% skimmed milk and 5% fetal bovine serum at room temperature for 1 h. Primary antibodies, including rabbit anti-WNT2B (1:1,000; Abcam), rabbit anti-phosphorylated β-CATENIN (1:1,000; Millipore), rabbit anti β-CATENIN (1:1,000; Abcam), anti-WNT5B (1:1,000; Abcam), and mouse monoclonal anti-β-ACTIN (1:5,000; Sigma), were diluted in blocking buffer for addition to the membranes, which were then incubated at 4°C overnight. Following 4 × 20 min washes, membranes were incubated with a secondary antibody conjugated to horseradish peroxidase (1:10,000; Jackson Laboratories, www.jacksonimmuno.com/) for 1 h at room temperature. The blots were visualized using the enhanced chemiluminescence advanced detection reagent ECL2 (Thermo Scientific) and a Fujifilm Imager (LAS-100; Fujifilm).

### Enzyme-Linked Immunosorbent Assay

The hMSC were grown for 5–6 days in DMEM with 2% FCS on bMP-coated flasks in the presence or absence of either human recombinant TGFβ1 (50 ng/mL) or FTRI to induce photoreceptor differentiation as indicated above [3]. Supernatants were collected and used for Enzyme-Linked Immunosorbent Assay (ELISA) analysis for quantification of secreted DKK1 (R&D Systems), WNT2B, and WNT5B (CUSABIO) using the manufacturer's instructions.

### Immunocytochemistry

The hMSC were grown for 7 days in DMEM supplemented with 2% FCS on bMP-coated glass coverslips placed in 24-well plates in the presence or absence of TGFβ1 and/or inhibitors. After 6–7 days cells were fixed in 4% PFA for 20 min. Slides were blocked for 1 h at room temperature using Roche blocking reagent (0.5% Blocking Solution; Roche Applied Science). Primary antibodies against rabbit anti-NR2E3 (1:50; Millipore), rabbit anti-recoverin (1: 250; Millipore) were diluted in blocking reagent and incubated overnight at 4°C. Primary antibody labeling was detected using donkey anti-rabbit or anti-mouse antibodies labeled with AlexaFluor 488 (1:500, Molecular Probes; Invitrogen) for 2 h at room temperature. DAPI was used to counterstain the cell nuclei, and slides were mounted with VECTASHIELD (VECTASHIELD; Vector Laboratories). Fluorescent images were captured with identical exposure times using a Zeiss LSM710 confocal microscope and identically processed using Carl Zeiss Zen imaging software (Carl Zeiss Microscopy GmbH).

### Statistical analysis

For PCR and western blots, the integrated optical density of each band was calculated using ImageJ software (ImageJ v3.9u; NIH). The optical density of each band was normalized by dividing the optical density of the sample by the optical density of its corresponding control gene (β-actin) band. Histograms were generated to represent the pixel intensities of each band. For ELISA, a standard curve was plotted of diluted standard solutions for each experiments (DKK1, WNT2B, and WNT5B) using Curve Expert 1.3 software. The concentration of proteins in each of the samples was identified by extrapolation to the generated curve. Statistical analysis of all results was carried out using Graphpad Prism 5 software. Statistical differences were calculated using paired Student's *t*-test or one-way repeated measures ANOVA. The standard error of the mean was plotted as error bars on bar charts and a probability of <0.05 was considered to be significant.

## Results

### TGFβ1 modulates the expression of components of the canonical and noncanonical Wnt signaling pathway in hMSC

**FIG. 1.**
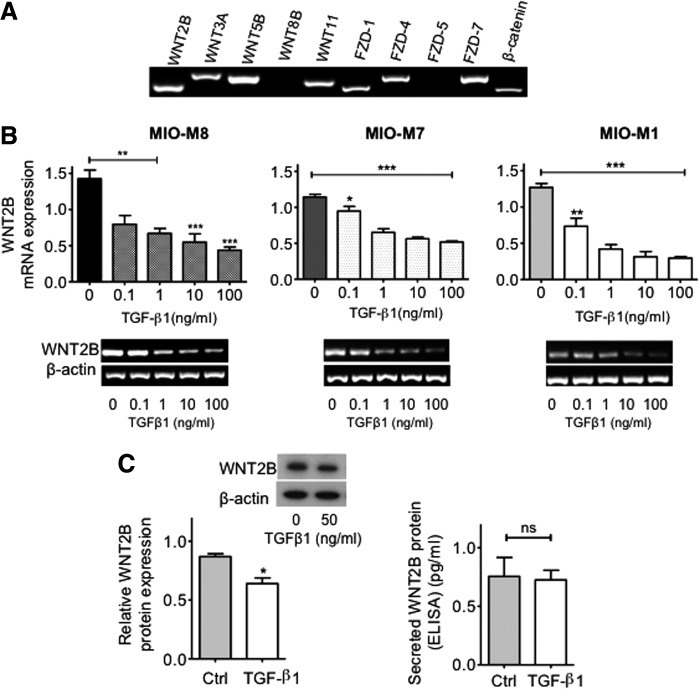
Expression of mRNA coding for molecules of the Wnt signaling pathway in human Müller stem cells (hMSC) and modulation of *WNT2B* expression by transforming growth factor-β (TGFβ1). **(A)** hMSC express mRNA coding for various components of the canonical and noncanonical Wnt signaling pathway. **(B)** TGFβ1 downregulation of the expression of mRNA coding for *WNT2B* occurred in a dose–response manner in three different hMSC lines (MIO-M8, MIO-M7, and MIO-M1) after 7 days culture with concentrations of this cytokine ranging between 0.1 and 100 ng/mL. Histograms represent the mean ± standard error of the mean (SEM) from UV spectrophotometer readings of gel bands. Representative bands are shown *below* the histograms; *n* = 3–4. ANOVA test, *P < 0.05; **P < 0.01; ***P < 0.001. **(C)** A significant decrease in the expression of WNT2B protein was observed by western blot analysis of lysates from cells cultured with 50 ng/mL of TGFβ1. Histograms represent the mean ± SEM of the relative optical density readings of gel bands. Representative bands are shown *above* the histograms; *n* = 3. Student's *t*-test; *P < 0.05. Minimally detectable levels of secreted WNT2B examined by Enzyme-Linked Immunosorbent Assay (ELISA) methods were observed in supernatants of cells cultured in the presence or absence of TGFβ1, and no differences between the two conditions were observed; *n* = 3. Student's *t*-test; ns, not significant.

TGFβ signaling through smad2/3 has been demonstrated to regulate photoreceptor regeneration in the adult zebrafish [10], while the canonical Wnt signaling pathway has been shown to regulate proliferation and differentiation of Müller glia-derived progenitors [22,23]. On this basis we first investigated whether hMSC expressed molecular components of the Wnt signaling pathway, and whether TGFβ, which is upregulated during retinal gliosis [24], had any effect on the expression of these molecules. RT-PCR analysis showed that hMSC express mRNA coding for *WNT2B*, *WNT3A*, *WNT5B*, *WNT11*, *FZD1*, *FZD4*, *FZD7*, and *β-CATENIN* under baseline conditions. Transcripts for WNT8B and FZD5 were not detected despite the use of three different primers and variations in assay parameters (Fig. 1A). Three different hMSC cell lines named, MIO-M8, MIO-M7, and MIO-M1, when cultured with various concentrations of TGFβ1 for 7 days showed that mRNA expression coding for the *WNT2B* ligand consistently decreased in a dose–response manner (Fig. 1B). As compared with the controls, concentrations as low as 0.1 ng/mL reached significant differences in all the three cell lines. Increasing log_10_ concentrations between 1 and 100 ng/mL of TGFβ1 induced a further decrease (*P* < 0.001 for all the cell lines examined) in the expression of this gene, without showing significant differences among them (Fig. 1B). Corresponding to that seen with mRNA expression, western blot analysis of MIO-M1 cells cultured with 50 ng/mL of TGFβ1 showed a significant decrease (*P* < 0.05) in intracellular WNT2B protein levels as compared with cells cultured in medium alone. Interestingly, the levels of WN2B ligand present in culture supernatants were minimally detected in both control and TGFβ1-treated cells (below 1 pg/mL) and there were no differences between the two conditions (Fig. 1C).

**FIG. 2.**
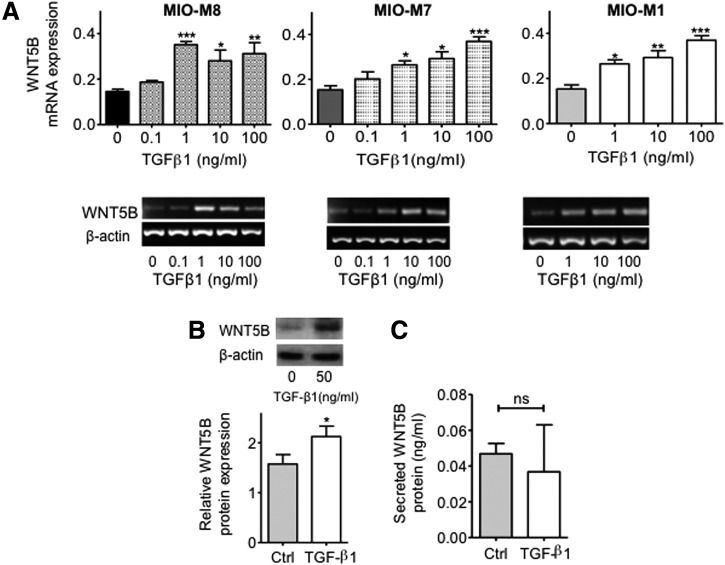
TGFβ1 modulation on the expression of WNT5B in hMSC. **(A)** TGFβ1-induced upregulation of the expression of mRNA coding for *WNT5B* occurred in a dose–response manner in three different hMSC lines examined (MIO-M7, MIO-M8, and MIO-M1). Histograms represent the mean ± SEM from UV spectrophotometer readings of gel bands. Representative bands are shown *below* the histograms; *n* = 4. ANOVA test, *P < 0.05; **P < 0.01; ***P < 0.001. **(B)** Western blot analysis of cell lysates from hMSC showed a marked increase of intracellular protein in cells treated with TGFβ1 at a concentration of 50 ng/mL as compared with control cells. Histograms represent the mean ± SEM of the relative optical density readings of gel bands. Representative bands are shown *above* the histograms; *n* = 4. Student's *t*-test, *P < 0.05. **(C)** Minimally detectable levels of secreted WNT5B, as examined by ELISA, were observed in supernatants of cells cultured in the presence or absence of TGFβ1, and no differences between the two conditions were observed; *n* = 3. ns, not significant.

In contrast to the downregulation of *WNT2B* mRNA caused by TGFβ1 in hMSC, mRNA expression of the *WNT5B* ligand was consistently increased by TGFβ1 in a dose–response manner (Fig. 2A). Although concentrations of 0.1 ng/mL of TGFβ1 did not cause significant changes in gene expression, increasing log_10_ concentrations ranging between 1 and 100 ng/mL of TGFβ1 induced a significant increase in the expression of *WNT5B* mRNA (*P* < 0.05 for MIO-M8; *P* < 0.001 for MIO-M7 and MIO-M1). In agreement with the mRNA findings, culture of hMSC with TGFβ1 caused a significant increase (*P* < 0.05) in the intracellular levels of WNT5B protein as compared with cells cultured in medium alone (Fig. 2B). Similarly to that seen with the levels of secreted WNT2B in the culture supernatant, secreted WNT5B protein was minimally detected in both control and TGFβ1-treated cells and there were no difference between the two conditions (Fig. 2C).

**FIG. 3.**
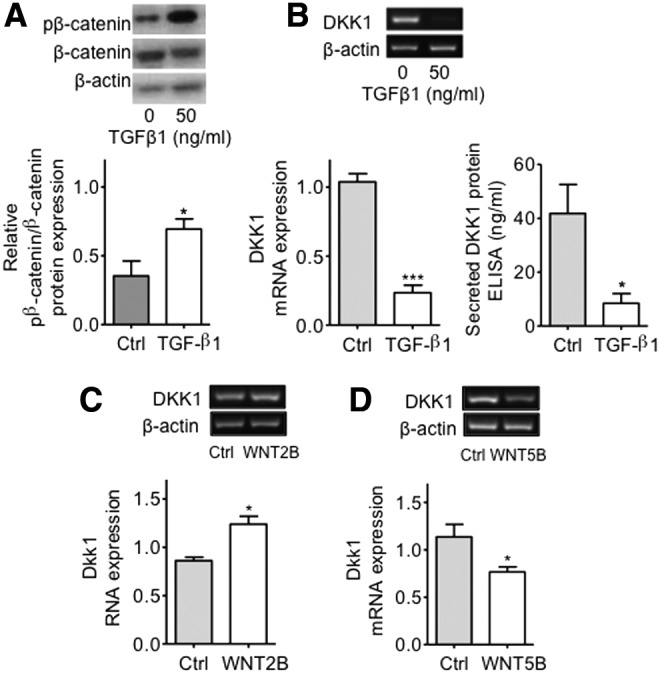
Modulation of pβ-catenin and DKK1 protein expressions by TGFβ1 and effect of exogenous WNT2B and WNT5B ligands on *DKK1* mRNA expression. **(A)** Western blot analysis revealed that culture of hMSC with 50 ng/mL of TGFβ1 induced a significant upregulation of the ratio of phospho-β-catenin/β-catenin. Histograms represent the mean ± SEM of the relative optical density readings of gel bands. Representative bands are shown *above* the histograms; *n* = 5. Student's *t*-test, *P < 0.05. pβ-catenin=phospho-β-catenin. **(B)** TGFβ1 caused a significant decrease in *DKK1* mRNA expression in hMSC as revealed by RT-PCR analysis. Histograms represent the mean ± SEM from UV spectrophotometer readings of gel bands. Representative bands are shown *above* the histograms; *n* = 8. Student's *t*-test; ***P < 0.001. Secreted DKK1 protein levels as determined by ELISA methods were significantly decreased in culture supernatants of cells treated with 50 ng/mL of TGFβ1 as compared to controls; *n* = 4. Student's *t*-test; *P < 0.05. **(C)** Exogenous addition of recombinant WNT2B into the culture medium induced a significant upregulation of *DKK1* mRNA in hMSC; *n* = 4. Student's *t*-test, *P < 0.05. **(D)** Addition of recombinant WNT5B to cells in culture caused a significant downregulation of *DKK1* mRNA expression; *n* = 4. Student's *t*-test, *P < 0.05. RT-PCR, reverse transcription-polymerase chain reaction.

Examination of the ratio of phosphorylated β-catenin over β-catenin protein expressions showed that the levels of phosphorylated β-catenin (which indicates that β-catenin is targeted for degradation) were increased by TGFβ1 in hMSC (*P* < 0.05) (Fig. 3A). Furthermore, TGFβ1 caused a significant decrease in the expression of mRNA coding for the canonical Wnt-signaling target *DKK1* (*P* < 0.001) and secreted DKK1 protein (*P* < 0.05) compared to cells cultured with medium alone (Fig. 3B). To test the effect of the WNT2B and WNT5B ligands on Wnt signaling in these cells, we cultured hMSC in the presence or absence of these two ligands and observed that while recombinant WNT2B significantly increased the levels of *DKK1* mRNA (*P* < 0.05) (Fig. 3C), recombinant WNT5B markedly decreased the mRNA levels of this target gene in hMSC (*P* < 0.05) (Fig. 3D).

These findings indicate that different adult hMSC lines express various components of the Wnt signaling pathway and that TGFβ1 downregulates the canonical Wnt signaling ligand WNT2B as well as the active form of β-catenin, which are important for canonical Wnt signaling. Furthermore, upregulation of the noncanonical Wnt ligand WNT5B by TGFβ1 may indicate the potential of this cytokine to inhibit the canonical Wnt signaling pathway.

### Canonical Wnt signaling components are upregulated by factors that induce photoreceptor differentiation of hMSC

**FIG. 4.**
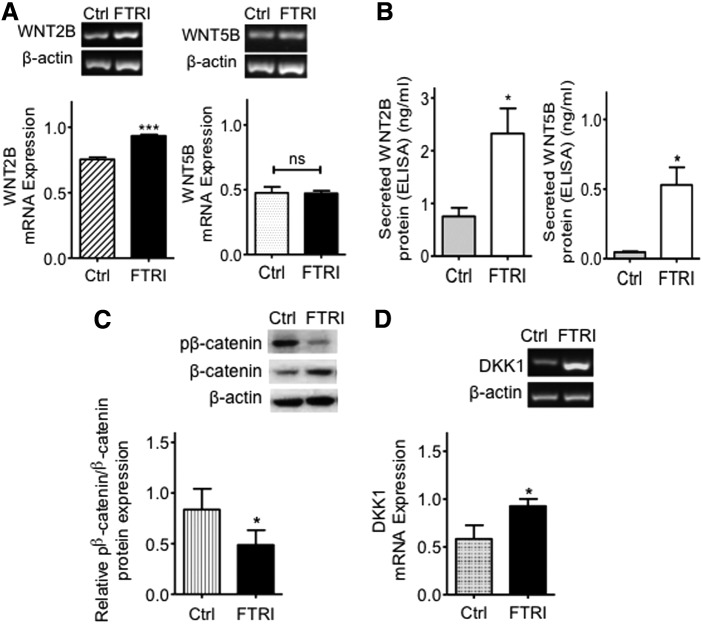
Induction of photoreceptor differentiation by FTRI causes changes in the expression of the Wnt signaling components WNT2B, β-catenin, and DKK1 in hMSC. **(A)** Culture of hMSC with FTRI for 7 days caused a significant increase in the expression of *WNT2B* mRNA, while no changes in the expression of WNT5B were observed in these cells. Histograms represent the mean ± SEM from UV spectrophotometer readings of gel bands. Representative bands are shown *above* the histograms; *n* = 5. Student's *t*-test, ***P < 0.001. **(B)** Quantification of the secreted ligands, as measured by ELISA, showed that both WNT2B and WNT5B were significantly increased in culture supernatants of hMSC treated with FTRI for 7 days; *n* = 3. Student's *t*-test, *P < 0.05. **(C)** Western blotting analysis showed that the ratio of phospho-β-catenin/β-catenin was decreased by FTRI treatment of hMSC. Histograms represent the mean ± SEM of the relative optical density readings of gel bands. Representative bands are shown *above* the histograms; *n* = 5. Student's *t*-test, *P < 0.05. pβ-catenin=phospho-β-catenin. **(D)** A significant increase in the expression of *DKK1* mRNA was observed in hMSC cultured with FTRI for 7 days; *n* = 3. Student's *t*-test, *P < 0.05. ns, not significant; FTRI, FGF2, taurine, retinoic acid and insulin-like growth factor type1.

Our recent studies have demonstrated that adult hMSC can be successfully differentiated into photoreceptors upon culture with FGF2, taurine, retinoic acid, and insulin-like growth factor type1 (FTRI) [3]. Given the importance of Wnt signaling in neural stem cell proliferation and differentiation, we examined the effect of FTRI on the Wnt signaling components of hMSC. Interestingly, conditions inducing photoreceptor differentiation of hMSC caused a significant increase in the expression of mRNA coding for the canonical Wnt signaling ligand *WNT2B* (*P* < 0.001), but did not modify the mRNA expression of *WNT5B* (*P* = 0.46) (Fig. 4A). A significant increase in the release of both WNT2B and WNT5B ligands into the culture supernatants was however observed when cells were cultured with FTRI (*P* < 0.05) (Fig. 4B). In addition, western blot analysis demonstrated that the ratio of phospho-β-catenin/β-catenin was significantly decreased in cells undergoing photoreceptor differentiation as compared to undifferentiated hMSC (*P* < 0.05) (Fig. 4C). Furthermore, when culturing cells under photoreceptor differentiating conditions, we also observed an increase in mRNA expression coding for the Wnt target *DKK1* (*P* < 0.05) (Fig. 4D).

These observations suggest that opposite to that seen with TGFβ1 alone, photoreceptor differentiation of hMSC induced by FTRI caused upregulation of the canonical Wnt signaling pathway in hMSC. This suggests that FTRI promotes signaling in hMSC through the Wnt canonical pathway.

### TGFβ1 inhibition of photoreceptor differentiation is associated with changes in the expression of Wnt ligands

Having shown that photoreceptor differentiation of hMSC promotes activation of the canonical Wnt signaling pathway and that TGFβ1 downregulates canonical Wnt signaling components while upregulating the noncanonical WNT5B ligand (Figs. 1 and 2), we examined whether TGFβ1 can modulate photoreceptor differentiation of hMSC by modifying the Wnt signaling pathway in these cells.

**FIG. 5.**
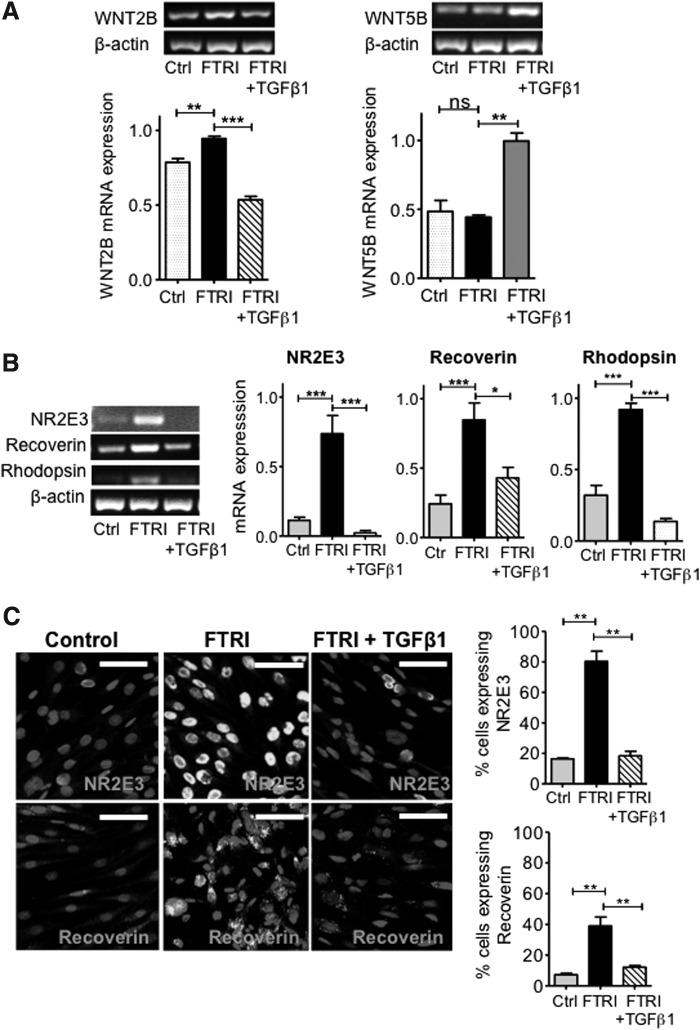
Inhibition of FTRI-induced photoreceptor differentiation of hMSC by TGFβ1. **(A)** Culture of MIO-M1 cells with FTRI caused an increase in *WNT2B* mRNA expression, but addition of TGFβ1 to the differentiation medium inhibited this increase; *n* = 4. ANOVA test, **P < 0.01; ***P < 0.001. FTRI alone did not modify *WNT5B* mRNA expression, but addition of TGFβ1 to the differentiation cocktail increased *WNT5B* mRNA expression (similar to that shown *above* with TGFβ1 alone). Histograms represent the mean ± SEM from UV spectrophotometer readings of gel bands. Representative bands are shown *above* the histograms; *n* = 3. ANOVA test, **P < 0.01; ***P < 0.001. **(B)** Addition of TGFβ1 to hMSC undergoing photoreceptor differentiation with FTRI inhibited the mRNA expression of *NR2E3*, recoverin, and rhodopsin as compared with FTRI alone; *n* = 5–8. ANOVA test, *P < 0.05; ***P < 0.001. **(C)** Immunostaining for NR2E3 and recoverin confirmed that while FTRI alone caused a marked increase in the expression of this photoreceptor protein, addition of TGFβ1 to hMSC cultured with FTRI caused inhibition of photoreceptor differentiation (Alexa 488, fluorescent cells). Cell nuclei counterstained with DAPI (non-fluorescent cell structures). Scale bars 50 μm. Histograms on the *right* represent the proportion of cells immunostaining for each of the markers following 7-day culture under the different conditions; *n* = 3. ANOVA test, **P < 0.01.

Addition of TGFβ1 to hMSC cultured in the presence of FTRI caused a significant decrease (*P* < 0.001) in *WNT2B* mRNA expression, as compared to hMSC cultured with FTRI alone (Fig. 5A). Cells cultured under differentiating conditions in the presence of TGFβ1 also showed a significant increase in *WNT5B* mRNA expression (*P* < 0.01) as compared to cells cultured in the presence of FTRI alone (Fig. 5A). These results suggest that by modifying the expression of the Wnt ligands WNT2B and WNT5B, TGFβ1 inhibits the effect of FTRI on hMSC. To assess whether this inhibitory effect was reflected on the ability of these cells to differentiate into photoreceptors, TGFβ1 was added to hMSC cultured with FTRI. Under photoreceptor differentiating conditions, TGFβ1 caused a significant downregulation of mRNA coding for the photoreceptor markers *NR2E3* (*P* < 0.001), *RECOVERIN* (*P* < 0.05), and *RHODOPSIN* (*P* < 0.001) as compared to hMSC cultured with FTRI alone (Fig. 5B). This was confirmed by a decrease in the number of cells expressing *NR2E3* (*P* < 0.01) and *RECOVERIN* (*P* < 0.01) when cells were cultured with FTRI in the presence of TGFβ1, as compared to cells cultured with FTRI alone (Fig. 5C).

**FIG. 6.**
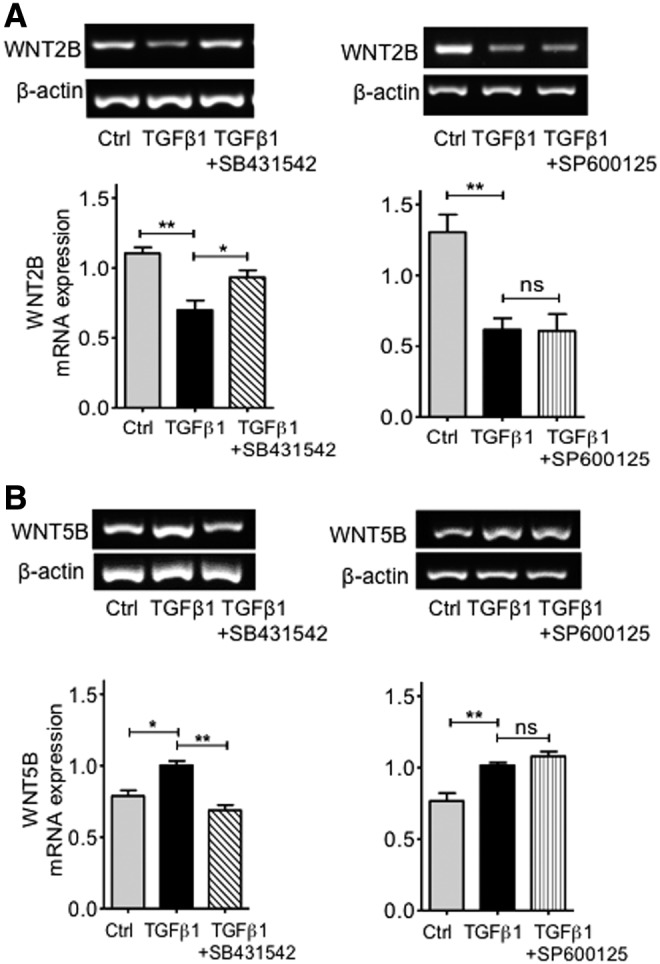
Effect of TGFβ1 inhibitors on the expression of the Wnt signaling ligands WNT2B and WNT5B by hMSC. **(A)** Addition of the TGFβ type I receptor (ALK5) inhibitor SB431542 (10 μM) to cells cultured with TGFβ1 antagonized the inhibitory effects of this cytokine on *WNT2B* mRNA expression; *n* = 5. ANOVA test; *P < 0.05; **P < 0.01. In contrast, addition of the JNK inhibitor SP600125 (20 μM) to cells cultured in the presence of TGFβ1 did not modify the effect of this cytokine on *WNT2B* gene expression. Histograms represent the mean ± SEM from UV spectrophotometer readings of gel bands. Representative bands are shown *above* the histograms; *n* = 3. ANOVA test, **P < 0.01. **(B)** While the ALK5 inhibitor SB431542 antagonized the upregulation of *WNT5B* mRNA by TGFβ1; *n* = 3. ANOVA test; *P < 0.05; **P < 0.01, the JNK inhibitor SP600125 did not modify the effects of this cytokine on the expression of this ligand gene; *n* = 4. ANOVA test, **P < 0.01; ns, not significant.

To assess whether inhibition of TGFβ signaling may modify the effect of this cytokine on the expression of *WNT2B* and *WNT5B*, we inhibited components of the TGFβ signaling pathway using the ALK5 receptor inhibitor SB431542 and the JNK inhibitor SP600125. As previously observed, Figure 6A shows that TGFβ1 alone caused a significant decrease in the expression of the *WNT2B* mRNA as compared with control cells (*P* < 0.01). However, addition of SB431542 markedly inhibited this effect (*P* < 0.05) (Fig. 6A). Unlike that seen with the ALK5 inhibitor, addition of the JNK inhibitor SP600125 did not cause any effect on the downregulation of *WNT2B* mRNA by TGFβ1 (*P* = 0.91) (Fig. 6A). This suggests that mRNA downregulation of the Wnt signaling ligand *WNT2B* by TGFβ1 is caused by activation of the SMAD2/3 signaling cascade. Similarly, the increase in *WNT5B* expression induced by TGFβ1 alone (*P* < 0.01) was inhibited by addition of SB431542 (*P* < 0.01) (Fig. 6B). This contrasts with the lack of inhibitory effect by JNK (SP600125) inhibitors (*P* = 0.14) (Fig. 6B). These observations suggest that the TGFβ1-induced upregulation of *WNT5B* expression in hMSC is also dependent of SMAD2/3 signaling, but independent of JNK transcription signaling. Taken together, these results suggest that TGFβ1 may regulate hMSC photoreceptor differentiation by modifying the ligands WNT2B and WNT5B of the Wnt signaling pathway.

### The canonical Wnt signaling is required for photoreceptor differentiation of hMSC

**FIG. 7.**
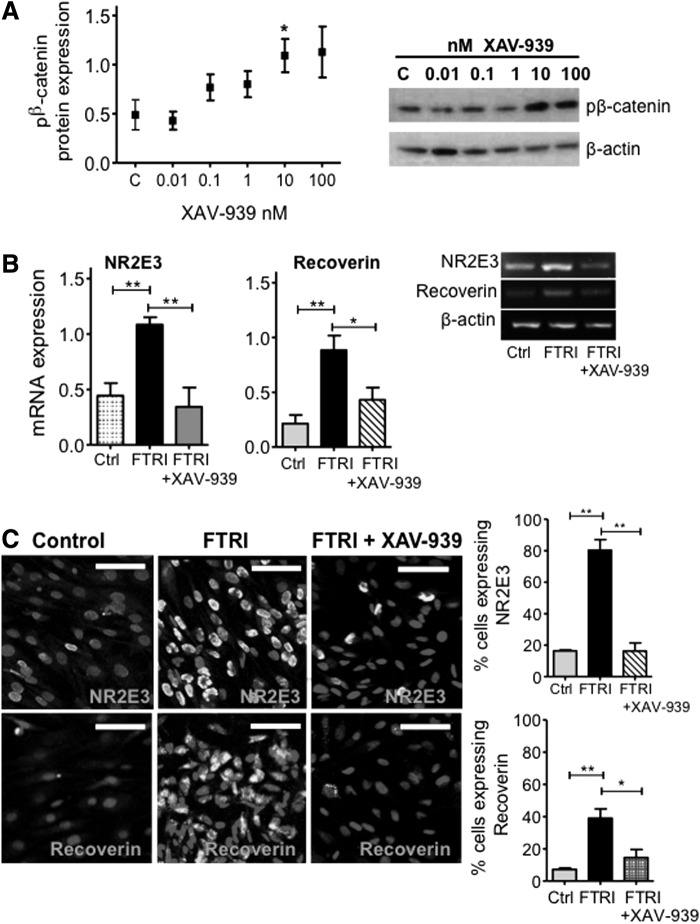
Effect of inhibition of *β-CATENIN* on the photoreceptor differentiation of hMSC. **(A)** Dose–response increase in p*β-CATENIN* expression by hMSC cultured with concentrations of XAV939 ranging between 0.01 and 100 nM. Western blot bands on the *right* show that XAV-939 significantly upregulated the expression of p*β-CATENIN* protein at concentrations of 10 and 100 nM; *n* = 4. Student's *t*-test, *P < 0.05. pβ-catenin=phospho-β-catenin. **(B)** Addition of XAV-939 to hMSC cultured with FTRI inhibited the differentiation of these cells into photoreceptors, as judged by the expression of mRNA coding for *NR2E3* and *RECOVERIN*. Histograms on the *left* represent the mean ± SEM from UV spectrophotometer readings of gel bands. Representative bands are shown on *right* of the histograms; *n* = 5–7. ANOVA test; *P < 0.05; **P < 0.01. **(C)** Confocal images confirmed that addition of XAV-939 to hMSC cultured in the presence of FTRI caused a decrease in the expressions of NR2E3 and recoverin, which is upregulated by FTRI alone (Alexa 488, fluorescent cells). Cell nuclei counterstained with DAPI (non-fluorescent cell structures). Scale bars 50 μm. Histogram represents the percentage of cells stained with NR2E3 following 7 days culture under the different conditions; *n* = 3. ANOVA test; *P < 0.05; **P < 0.01.

Following observations that FTRI, which induces photoreceptor differentiation of hMSC, causes upregulation of canonical Wnt signaling components in these cells, we examined whether Wnt signaling is required for hMSC photoreceptor differentiation. We first tested the effect of the tankyrase inhibitor XAV-939, known to effectively block β-catenin by stabilizing axin [25], on undifferentiated cells, and observed that increasing log_10_ concentrations of this compound caused a gradual increase in the expression of phosphorylated β-catenin. Although significant increase was only observed with concentrations above 10 nM (*P* < 0.05) (Fig. 7A). Addition of XAV-939 (10 nM) to hMSC cultured under photoreceptor differentiating conditions caused a significant inhibition of the effect of FTRI on the mRNA expression of the photoreceptor markers *NR2E3* (*P* < 0.01) and *RECOVERIN* (*P* < 0.05) (Fig. 7B). This inhibition was further confirmed by immunocytochemical analysis, which also demonstrated a significant decrease in the number of cells expressing NR2E3 (*P* < 0.01) and *RECOVERIN* (*P* < 0.05) proteins in hMSC cultured with FTRI in the presence of XAV-939 (*P* < 0.01) (Fig. 7C). Taken together, these results suggest that signaling through the canonical Wnt pathway precedes the activation of proneural factors involved in the differentiation of hMSC into photoreceptors.

## Discussion

Müller glia, which are responsible for the spontaneous retina regeneration observed in zebrafish [10,26], have also shown limited regenerative ability in early postnatal life in small vertebrates [27,28]. Although a population of Müller glia isolated from the adult human retina exhibit stem cell characteristics in vitro [2], there is no indication that these cells have any regenerative ability in vivo. There is much evidence for the roles of TGFβ and Wnt signaling in the mediation of cellular processes regulating Müller glia differentiation in the zebrafish retina [10,29], and rodent retina [30], as well as in the patterning of the eye during embryonic development [31,32]. However, very little is known of the role of these factors in the regulation of progenicity and neural differentiation of Müller glia in the adult human eye.

**FIG. 8.**
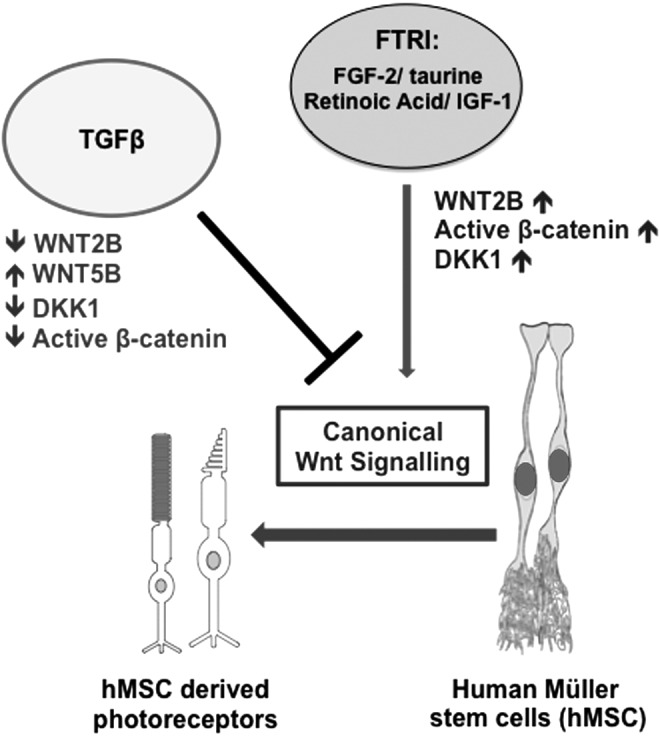
TGFβ1 inhibits the canonical Wnt signaling pathway necessary for the photoreceptor differentiation of hMSC in vitro. Schematic illustration summarizing the interactions of FTRI, WNT2B, WNT5B, TGFβ1, and DKK1 in hMSC. FTRI, which induces photoreceptor differentiation of hMSC, activated the canonical Wnt signaling pathway in these cells. Addition of TGFβ1 to cells cultured with FTRI caused inhibition of the canonical Wnt signaling and consequently inhibited the photoreceptor differentiation of hMSC in vitro.

Activation of the TGFβ and Wnt signaling pathways require the expression of specific receptors on the cell surface, and as previously shown, mammalian Müller glia express TGFβ and Wnt receptors and their ligands [19,33–35], for which it is possible that activation of these pathways may trigger the neurogenic properties of human Müller glia as observed in other species. As illustrated in Figure 8, our results showed that TGFβ caused in vitro downregulation of the canonical Wnt signaling pathway in hMSC. This was demonstrated by a decrease in the expression of WNT2B, DKK1, and active β-CATENIN in cells cultured with this cytokine. In contrast, FTRI, which induces photoreceptor differentiation of hMSC [3], upregulated the expression of genes and proteins associated with the canonical Wnt signaling pathway. More important, addition of TGFβ1 to hMSC cultured with FTRI resulted in inhibition of the photoreceptor differentiation. These findings suggest that as seen in the zebrafish and early postnatal life in small vertebrates, photoreceptor differentiation of hMSC requires activation of canonical Wnt signaling and that by modulating this pathway, TGFβ1 may control the neurogenic ability of these cells in the human eye.

It has been documented that loss of TGFβ or Wnt5a (which shares 80% protein sequence with human WNT5B (www.omim.org/entry/606361) in mouse mammary cells, results in increased Wnt/β-catenin activity [36], and that TGFβ signaling in mouse embryos blocks the canonical Wnt pathway, leading to inhibition of midbrain development [37]. In addition, overexpression of *wnt5b* in zebrafish causes downregulation of the canonical Wnt target *axin2*, with consequent inhibition of tail fin regeneration [38]. Therefore, the present findings that inhibition of photoreceptor differentiation by TGFβ1 in hMSC was accompanied by gene downregulation of *WNT2B* and upregulation of *WNT5B*, is in accordance with the reported functions of this ligand in promoting the activation of the canonical Wnt signaling pathway in other species. This suggests that by increasing WNT5B expression, TGFβ1 may potentiate its inhibitory effect that prevents photoreceptor differentiation of hMSC. That FTRI upregulates the expression of WNT2B strongly suggests that the effect of these factors on the photoreceptor differentiation of hMSC may be exerted through the activation of the canonical Wnt signaling pathway.

Regulation of intracellular functions by TGFβ involves the activation of SMAD proteins that translocate to the nucleus, where they regulate transcription, as well as SMAD-independent pathways such as those involving JNK and p38 activation [39]. We observed that SB431542, an inhibitor of ALK5 receptor, which selectively blocks the SMAD2/3-dependent pathway [40], antagonized the effect of this cytokine on the downregulation of WNT2B. Similarly, upregulation of WNT5B was antagonized by the ALK5 receptor inhibitor SB431542. This contrasted with the lack of effect of the JNK inhibitor on the modulation of both ligands by TGFβ1. These results suggest that modulation of the expression of WNT2B and WNT5B by TGFβ1 might be caused by SMAD signaling activation. Previous studies have shown that in the zebrafish regulation of Smad2/3 signaling in Müller glia is important for the proliferative and neurogenic response of these cells to retinal damage [10]. Hence, the present observations that known intracellular pathways of Müller cell differentiation observed in zebrafish are also active in human Müller glia in vitro suggest the existence of inhibitory mechanisms of these pathways in the adult human retina, which may prevent these cells from regenerating the retina in vivo. It also raises the prospects that if we can control these mechanisms in hMSC in vitro, we could potentially induce Müller glia to regenerate the retina in vivo.

Involvement of the canonical Wnt signaling pathway in the photoreceptor differentiation of hMSC in vitro is further supported by the present findings that inhibition of Wnt signaling by XAV-939 (which stabilizes axin and consequently targets β-CATENIN for degradation) in cells cultured with FTRI, prevented photoreceptor differentiation of these cells. Activation of canonical Wnt signaling is associated with the maintenance and proliferation of retinal progenitors in the embryonic chick and mouse retina [41], while laser injury in transgenic mice lacking the Wnt signaling regulator *Axin2*, induces amplification of Wnt signaling and generation of rhodopsin-positive cells from Müller glia [35]. Wnt signaling activation is also associated with Müller glia-mediated regeneration in the zebrafish [20], and continuous activation of this pathway after acute injury in larval zebrafish also promotes the generation of neuronal progenitors from Müller glia [29].

To summarize, we have demonstrated that differentiation of hMSC into photoreceptors in vitro is dependent on the activation of the canonical Wnt signaling pathway and that TGFβ, which is highly upregulated during gliosis [24], modifies Wnt signaling mechanisms in hMSC (Fig. 8). That hMSC express genes of the Wnt signaling pathway and that their activation regulates photoreceptor differentiation upon culture with differentiation factors may reflect their potential regenerative ability in vivo. Given that signaling cascades elicited by binding of TGFβ and Wnt ligands to their receptors involve cross talks of intracellular signaling pathways [42], it may be possible that regulation of TGFβ and Wnt signaling may have diversified during evolution to prevent uncontrolled growth and differentiation of human Müller glia in the adult retina. It may be also possible that factors released during inflammation and gliosis could inhibit the regenerative ability of these cells in vivo. On this basis, comparative investigations into mechanisms that control these pathways in zebrafish and human Müller glia may help to identify therapeutic targets that could be potentially used to promote endogenous regeneration of the human retina, and this merits further investigations.
